# Thirteen-Year Trajectories of Depressive and Anxiety Symptoms in Individuals with and without Migraine: Insights from the NESDA Cohort

**DOI:** 10.1192/j.eurpsy.2025.1378

**Published:** 2025-08-26

**Authors:** N. Van Veelen, N. Pelzer, B. Penninx, G. M. Terwindt, E. J. Giltay

**Affiliations:** 1 Neurology, Leiden University Medical Center, Leiden; 2 Public Health and Primary Care, Leiden University Medical Center, The Hague; 3 Psychiatry, Amsterdam UMC, Amsterdam; 4 Psychiatry, Leiden University Medical Center, Leiden, Netherlands

## Abstract

**Introduction:**

Migraine is strongly associated with depressive and anxiety disorders, as migraine patients have over a twofold higher risk of major depressive disorder and nearly a threefold higher risk of anxiety disorders compared to non-migraine individuals.

**Objectives:**

This study investigates the 13-year trajectories in individuals with a depression or anxiety disorder with and without comorbid migraine. Additionally, the time to recovery of the depressive or anxiety disorder was compared.

**Methods:**

The NESDA cohort consists of participants with an affective disorder at baseline, according to the Composite International Diagnostic Interview (CIDI). Depressive symptoms were measured using the Inventory of Depressive Symptomatology (IDS) and anxiety symptoms with the Beck Anxiety Inventory (BAI). The participants were divided at baseline into three subgroups: migraine, non-migrainous headache and non-headache. The individual symptom trajectories were evaluated over 13 years with linear mixed models. Additionally, the time to recovery of the affective disorders with or without migraine were compared with a Cox-proportional hazard analysis.

**Results:**

In total 1,448 participants had either a depressive or anxiety disorder or a combination of both at baseline, 327(23%) had additional comorbid migraine, 518(36%) non-migrainous headache. Participants with comorbid migraine consistently reported higher severity of depressive and anxiety symptoms than non-headache participants over the 13-year follow-up, with an estimated higher IDS sum score of 0.22 (p<0.001) and a higher BAI sum score of 0.28 (p<0.001) (see Figure 1 and 2). The time to recovery of an affective disorder was similar for migraine and non-headache individuals (HR: 1.13 (p=0.17)).

**Image:**

**Figure:**
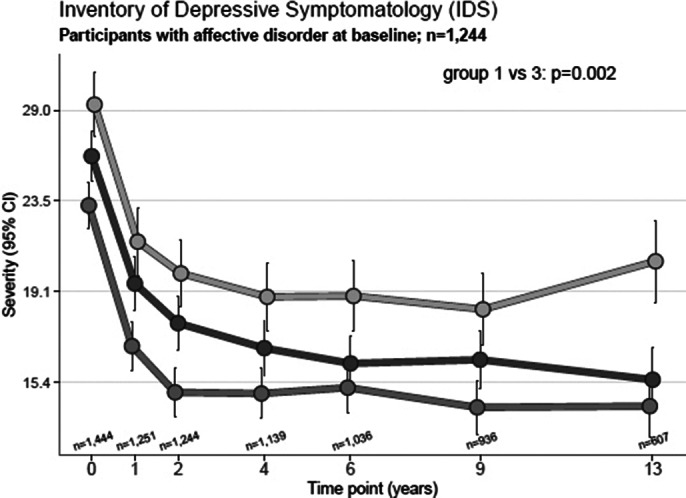

**Image 2:**

**Figure:**
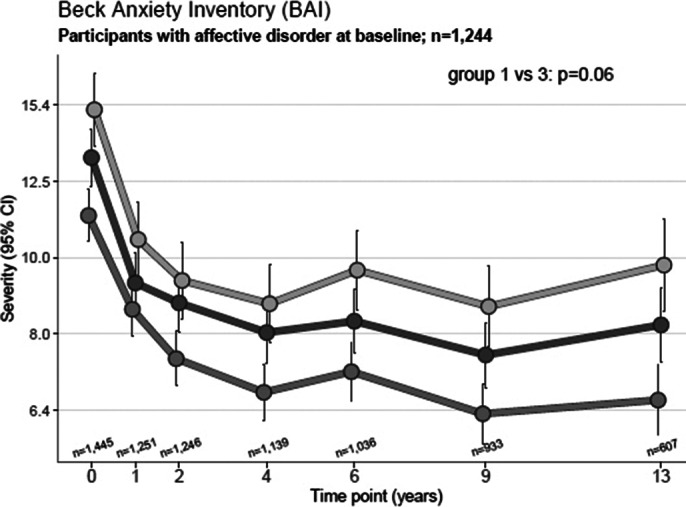

**Conclusions:**

In this cohort, individuals with comorbid migraine showed consistently higher severity of depressive and anxiety symptoms than non-headache individuals over 13 years. Recovery time from an affective disorder was similar for migraine and non-headache individuals.

**Disclosure of Interest:**

N. Van Veelen: None Declared, N. Pelzer Grant / Research support from: Independent support from the European Community, Dutch Heart Foundation, Dutch Research Council, Dutch Brain Foundation, Dioraphte, and the Clayco foundation, Consultant of: Consultancy support from Abbvie/Allergan, Lilly, Lundbeck, Novartis, Pfizer, Teva, B. Penninx: None Declared, G. Terwindt Grant / Research support from: Independent support from the European Community, Dutch Heart Foundation, Dutch Research Council, Dutch Brain Foundation, Dioraphte, and the Clayco foundation, Consultant of: Consultancy support from Abbvie/Allergan, Lilly, Lundbeck, Novartis, Pfizer, Teva, E. Giltay: None Declared

